# Cancer of Unknown Primary: A Case Report on the Recognition of Its Clinical Entity and Standard of Care Management

**DOI:** 10.7759/cureus.86451

**Published:** 2025-06-20

**Authors:** Alexander Lazzaro, Maya Srinivasan, Hussein Assi

**Affiliations:** 1 Medicine, Boston Medical Center, Boston University Chobanian and Avedisian School of Medicine, Boston, USA; 2 Hematology and Oncology, Boston Medical Center, Boston University Chobanian and Avedisian School of Medicine, Boston, USA

**Keywords:** cancer of unknown origin, cytokeratins, diagnostic mystery, immunohistochemistry (ihc), metastatic adenocarcinoma of unknown primary

## Abstract

Cancer of unknown primary (CUP) is a distinct and well-established clinical entity that encompasses a heterogeneous group of histologically proven cancers that present unique diagnostic and therapeutic challenges. This case describes a male patient in his late 60s who presented with nonspecific gastrointestinal complaints. A comprehensive workup, including clinical evaluation and various biochemical, imaging, and invasive histopathologic tests, led to the diagnosis of CUP following consultation with medical oncology specialists. A decision was made to forgo further invasive molecular tissue sampling in search of a primary malignant process in favor of initiating systemic chemotherapy. The patient possessed an unfavorable set of prognostic signs. Rapid clinical deterioration occurred the day after treatment initiation. A goals of care conversation led to a transition towards comfort measures only care, and the patient ultimately passed away. This case highlights the importance of non-oncologically trained providers recognizing CUP as its own entity to facilitate standard of care management. Future research is needed to establish benefits in clinical trials looking at molecular tumor profiling and site-specific therapies for this rare malignancy.

## Introduction

Cancer of unknown primary (CUP), a well-established clinical entity that is distinct and evolving, comprises a heterogeneous group of histologically proven, mainly epithelial cancers that have metastasized by the time of presentation and whose primary site has not been identified despite a typical diagnostic workup [1,2]. CUPs are hypothesized to exhibit distinct biologic features that allow for early and rapid metastatic spread from a primary tumor that is either too small or simply below thresholds for detection via conventional diagnostic methods [3].

CUP accounts for approximately 2% of all cancer diagnoses; the American Cancer Society (ACS) estimates that about 37,370 cases will be diagnosed in the United States in 2025 [4,5]. Rates have declined in recent years, likely due to enhanced capabilities of molecular tumor profiling [6]. As with other malignancies, traditional cancer-causing risk factors such as tobacco smoking, alcohol consumption, diabetes mellitus, and family history are also CUP risk factors; there remains insufficient evidence to conclude if any specific risk factor profile exists [7].

Common presenting clinical symptoms of CUP are non-specific, general complaints. Prevalence is roughly equal in men and women, with an average age of diagnosis of 60 [8]. Life expectancy is short, with a median survival of eight to 12 months from the time of diagnosis, dependent upon several prognostic factors [9]. Clinical evaluation comprises a comprehensive workup in search of a primary pathologic diagnosis [10] (Appendix A). Updated guidelines still favor empiric systemic chemotherapy, which is palliative in most settings, over the use of site-specific therapy, with recommendations to pursue available clinical trials [10]. There has been no substantial improvement in median survival times in recent years despite advancements in diagnostics [10].

Patients with CUP receive less treatment but use more health services, encompassing more tests, primary care consultations, and emergency room visits than patients with known primary malignancies [11]. The cost of overall CUP care is higher due to extensive diagnostic workups and non-site-specific treatment approaches [12]. Due to the relative rarity of the condition, the high morbidity and mortality, and potential for future management changes with expanded research, there is educational benefit to be gained from highlighting a case presentation of CUP. We present a case report of CUP in a patient who presented with non-specific gastrointestinal (GI) symptoms and whose course included varied inconclusive diagnostic tests prior to specialist consultation and correct diagnosis. Ultimately, rapid clinical deterioration occurred following systemic treatment initiation. The goal is to shed light on the distinct entity of CUP to facilitate a higher degree of diagnostic understanding amongst general, non-oncologic-trained providers and thus promote appropriate, resource-efficient workup and earlier treatment initiation, including referral to a clinical trial center if applicable.

## Case presentation

A male patient in his late 60s who was an immigrant to America, with a past medical history of alcohol and tobacco use disorder, presented to the emergency department with several weeks of inability to tolerate oral intake, nausea, vomiting, and constipation. The patient was admitted to the general medicine inpatient wards and was noted to have been hospitalized with the same symptoms one month prior, whereby he was diagnosed with an ileus of unclear etiology, managed conservatively, and discharged upon symptomatic improvement. Symptoms returned shortly thereafter and progressively worsened, prompting the current presentation. The patient’s vital signs and mentation were normal. The patient’s exam revealed hypoactive bowel sounds, moderate abdominal distension, and drainage of bilious fluid via a nasogastric tube that was placed in the emergency department. The first clinical impression was concerning for a mechanical bowel obstruction. The repeated constellation of symptoms and exam findings, coupled with the patient’s prior alcohol use disorder (now in remission) and 30+ pack-year tobacco smoking history, made a malignant etiology possible despite an unrevealing workup one month prior.

**Figure 1:**
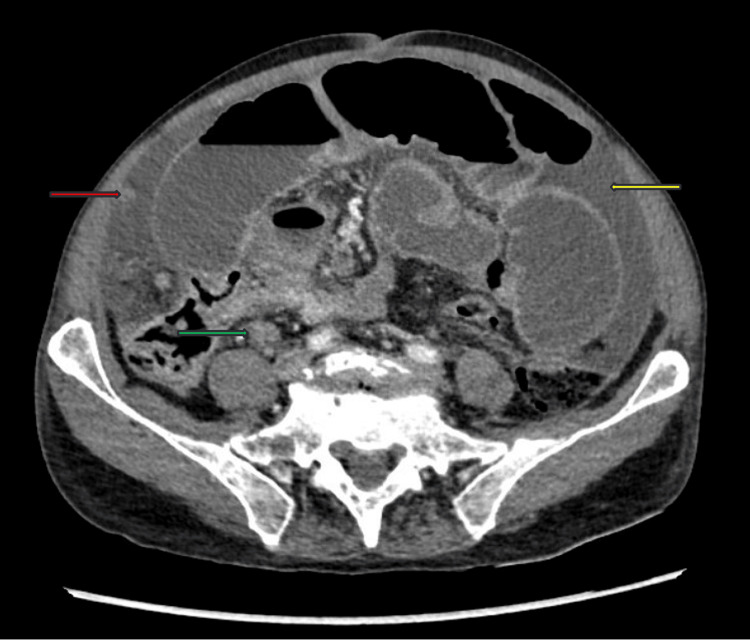
Abdominal CT scan on presentation Cross-sectional computed tomography (CT) image of the abdomen showing large-volume ascites (yellow arrow), mesenteric adenopathy in the right lower quadrant (green), and peritoneal thickening and nodularity (red). No abnormalities appreciated in intra-abdominal and intra-pelvic organs. Contiguous axial 1.25 mm images were obtained from the lung bases to the greater trochanters with 100 cc Isovue 370 intravenous contrast in the portal venous phase. Coronal, sagittal, and 3D maximum intensity projection (MIP) reconstructions were obtained.

Initial labs were notable for an elevated total bilirubin of 1.6 mg/dl and direct bilirubin of 0.6 mg/dl, and a decreased albumin of 2.9 g/dl. A complete blood count was normal. Results of computed tomography (CT) imaging of the abdomen and pelvis showed mild bilateral pleural effusions at the lung bases, nodular contours of the liver suggestive of cirrhosis, diffuse small bowel dilatation with mesenteric nodularity and lymphadenopathy, diffuse intraperitoneal free fluid, and areas of peritoneal thickening and omental nodularity suspicious for peritoneal carcinomatosis. There was a normal appearance of intrathoracic, abdominal, and pelvic organs otherwise (Figure 1). Higher suspicion for a metastatic oncologic process arose, with greatest concern for an underlying primary GI process. Also on the differential diagnosis was decompensated cirrhosis, due to prior social history and current exam and radiographic findings. There was low suspicion for an infectious process due to the patient’s afebrile state, normal white blood cell count, and lack of peritoneal signs on exam.

A diagnostic paracentesis was pursued to ascertain the etiology of ascites. 200 milliliters of clear yellow fluid were removed; results were positive for metastatic adenocarcinoma with CK20-/CK7+/CDX2+ immunophenotype, suggestive of upper GI or pancreatobiliary origin [13]. A carbohydrate antigen 19-9 (CA 19-9), a tumor marker often elevated in pancreatic and biliary cancers, and carcinoembryonic antigen (CEA), a biomarker elevated in many malignancies, were ordered to assist in diagnostic localization; the former resulted elevation at 85 units/ml (reference range: 0-37 units/ml), and the latter resulted in a normal level at 4.4 ng/ml (reference range: 0-4.9 ng/ml). The gastroenterology service was then consulted to perform an esophagogastroduodenoscopy (EGD) with endoscopic ultrasound (EUS), which showed mild nonspecific gastropathy, an atrophic pancreas, and moderate gallbladder sludge and multiple gallstones but no aberrant tissue or mass visualized to sample. A magnetic resonance imaging cholangiopancreatogram (MRCP) was pursued to better visualize the biliary and pancreatic systems. Adding to the diagnostic uncertainty, results showed choledocholithiasis with no acute cholecystitis, no gallbladder mass or detectable lesion, no pancreatic lesion, and no pancreatic ductal dilation. Colonoscopy was unable to be safely performed due to the presence of persistent ileus.

**Figure 2:**
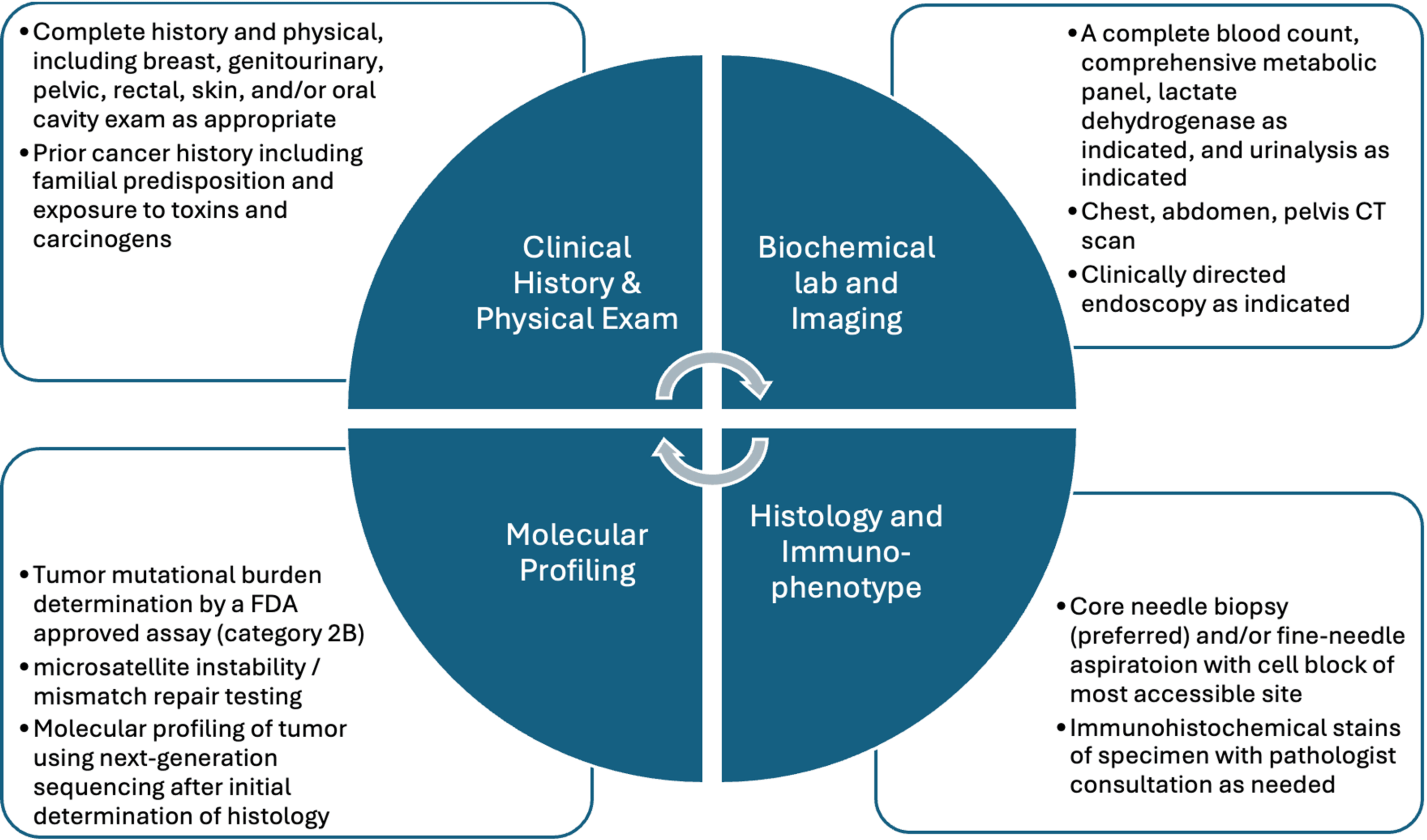
National Comprehensive Cancer Network® (NCCN®)-recommended diagnostic workup for cancer of unknown primary As a diagnosis of exclusion, a thorough initial evaluation and workup must be conducted to diagnose cancer of unknown primary. This should commence with a complete history and physical as a first line, followed by biochemical and imaging modalities, then histologic testing and immunophenotyping, and consideration of molecular profiling. Adapted with permission from the NCCN Clinical Practice Guidelines in Oncology (NCCN Guidelines®) for Occult Primary V.2.2025 [10]. © 2024 National Comprehensive Cancer Network, Inc. All rights reserved. The NCCN Guidelines® and illustrations herein may not be reproduced in any form for any purpose without the express written permission of NCCN. To view the most recent and complete version of the NCCN Guidelines, go online to NCCN.org. The NCCN Guidelines are a work in progress that may be refined as often as new significant data becomes available.

Without a clearly identified primary lesion on varied imaging and invasive diagnostic modalities, despite the positive immunohistochemistry findings of metastatic adenocarcinoma of likely upper GI or pancreatobiliary origin, the differential diagnosis remained broad. Cholangiocarcinoma and pancreatic adenocarcinoma were at the top of this differential. Medical oncology was consulted to guide further management steps. The case was discussed amongst a group of multidisciplinary oncologic practitioners. Further diagnostic investigation was halted as a diagnosis of CUP, specifically adenocarcinoma of unknown primary, was established. No characteristic biochemical or imaging findings nor definitive cytologic or histologic results indicated a primary etiology following comprehensive workup (Figure 2) [10]. The key to the correct diagnosis and management of CUP is recognition of its established clinical entity. CA 19-9, while mildly elevated, is an established biomarker for both pancreatic ductal adenocarcinoma and cholangiocarcinoma with insufficient sensitivity and specificity to be reliably used as a routine screener, let alone be utilized for establishing a diagnosis [14,15]. The diagnostic paracentesis yielded the presence of metastatic adenocarcinoma cells without characteristic cells indicative of origin. In the absence of an imaging correlate indicative of a primary lesion, the decision to biopsy a peritoneal nodule was foregone, as it was felt unlikely to provide novel information and change management.

In a goals of care conversation, the patient expressed a strong desire to pursue treatment. The decision was made to offer FOLFOX (folinic acid, 5-fluorouracil, and oxaliplatin). The patient remained unable to tolerate oral intake. The nasogastric tube remained in place for decompression. Total parenteral nutrition was initiated. Functional status was limited and progressively worsened. Risks and benefits were discussed, including an overall poor prognosis and the unlikelihood of treatment leading to a meaningful response to the point of resolving ileus and obstructions in the future. Unfortunately, the patient experienced acute hypoxic respiratory failure, an episode of unresponsiveness, and shock of unknown etiology on the third day of the first chemotherapy cycle, necessitating transfer to the medical intensive care unit. A new goals of care conversation was held with the patient’s healthcare proxy. The decision was made to withhold further treatment and transition to Do Not Attempt Resuscitation (DNAR) and comfort measures only (CMO) status. The patient passed away the following day.

## Discussion

The pursuit of further diagnostic studies, i.e., endoscopic retrograde cholangiopancreatography (ERCP) or fine-needle aspiration (FNA), is unlikely to provide additional information in search of an occult pancreatobiliary primary without a CT or EUS correlate. Imaging, outside of routine CT of the chest, abdomen, and pelvis, and invasive diagnostic tests should be directed towards investigating specific symptoms or signs [1]. Further attempts at metastatic tissue sampling without imaging correlation would not alter the treatment course of already known metastatic adenocarcinoma previously obtained by paracentesis, and thus risks of invasive testing outweigh any potential benefit [1]. No prospective data exists that suggests positron-emission tomography (PET) scans substantially add to the information obtained by CT scans for cases of adenocarcinoma of unknown primary. Lastly, autopsy studies have demonstrated that 70% of cases of CUP remain undiagnosed, further validating cessation of further broad testing [1].

CUP can be classified into four major subtypes, with the most frequent being well or moderately differentiated adenocarcinoma (50%), followed by poorly differentiated or undifferentiated adenocarcinoma (30%), squamous cell carcinoma (15%), and undifferentiated neoplasms (5%), including neuroendocrine tumors of unknown primary [1]. In patients with differentiated adenocarcinoma or undifferentiated carcinoma, 70% of patients have been found to have chromosomal instability (aneuploidy), a finding similar to that reported for adenocarcinomas of known histogenesis [16]. Overexpression of c-myc, ras p21, and c-erB-B2 p185 oncoproteins was identified in 96%, 92%, and 65% of cases, respectively, in one large case series [17].

Immunohistochemical studies, including the immunoperoxidase technique using a series of monoclonal or polyclonal antibodies to enzymes, structural tissue components (i.e., cytokeratins; CK), hormonal receptors, hormones, oncofetal antigens, or other substances, are useful for cell-type determination and pathologic diagnosis in patients with occult primary tumors [18]. In particular, the cytokeratins CK7 and CK20, two intermediate filament proteins whose expression patterns are closely associated with tissue differentiation and lineage within epithelial cells, are the two most commonly used immunostains for differentiation of occult primary tumors into subsets of carcinomas [18]. While the NCCN does not necessarily discourage next-generation sequencing (NGS) for unknown primary tumors, at present, they state there is no prospective data to support the use of biomarker-driven therapy beyond FDA-approved therapeutics approved for tumor-agnostic aberrations [10].

Patients with CUP may present with an unfavorable or favorable set of prognostic signs [19]. Our patient possessed unfavorable features, including both male gender and the presence of nonpapillary malignant ascites (adenocarcinoma) [19]. Treatment and ensuing response vary by histology and favorability of subtype; localized adenocarcinoma of unknown primary should be treated according to the most likely primary site. A previous study identified that patients with a favorable CUP subtype experienced superior overall survival compared to those with unfavorable subtypes (36.6 months vs. 3.8 months) [20]. Current NCCN Clinical Practice Guidelines in Oncology (NCCN Guidelines®), based on all available data, recommend mostly platinum-based systemic chemotherapy regimen approaches as the first-line option in our case, with the exception being FOLFIRI (irinotecan, 5-fluorouracil, and leucovorin) which can be considered in cases of presumed GI primary site. Following NCCN Guidelines® and risk profile for our patient, no molecular profiling was sent, and a platinum-based systemic chemotherapy regimen was pursued, ultimately with unfortunate results [10].

## Conclusions

The diagnosis of CUP often includes a comprehensive yet protracted workup in search of a primary oncologic process. Immunohistochemistry testing is essential for narrowing down likely organs of histologic etiology. Guideline-recommended treatment still favors systemic chemotherapy, although there is a growing body of evidence suggesting future utilization of molecular tumor profiling for enhanced diagnostic accuracy to facilitate more site-specific therapy. Earlier recognition of this disease entity, which is known for being highly morbid, allows for earlier referral to specialist oncology services and treatment start times. Future considerations should be held to discuss, in shared decision-making with patients, referral to CUP clinical trial centers. In retrospect, a biopsy of a peritoneal nodule to send tissue for genomic profiling and/or genomic profiling with a blood-based assay could have been used to identify potential actionable genetic alterations. This case highlights the need for clinicians, especially those non-oncologically trained, to recognize CUP as an independent entity to enable standard of care management and allow for consideration of patient referral to a specialty center and enrollment in a clinical trial if willing.

## Appendices

Appendix A

Reference 10: Referenced with permission from the NCCN Clinical Practice Guidelines in Oncology (NCCN Guidelines®) for Occult Primary V.2.2025. © National Comprehensive Cancer Network, Inc. 2024. All rights reserved. Accessed June 9, 2025. To view the most recent and complete version of the guideline, go online to NCCN.org. NCCN makes no warranties of any kind whatsoever regarding their content, use or application and disclaims any responsibility for their application or use in any way.
